# Diagnostic gene biomarkers for predicting immune infiltration in endometriosis

**DOI:** 10.1186/s12905-022-01765-3

**Published:** 2022-05-18

**Authors:** Chengmao Xie, Chang Lu, Yong Liu, Zhaohui Liu

**Affiliations:** Department of Gynecology, Beijing Obstetrics and Gynecology Hospital, Capital Medical University, Beijing Maternal and Child Health Care Hospital, Beijing, 100026 China

**Keywords:** Endometriosis, GEO, Immune infiltration, CIBERSORT, Biomarker

## Abstract

**Objective:**

To determine the potential diagnostic markers and extent of immune cell infiltration in endometriosis (EMS).

**Methods:**

Two published profiles (GSE7305 and GSE25628 datasets) were downloaded, and the candidate biomarkers were identified by support vector machine recursive feature elimination analysis and a Lasso regression model. The diagnostic value and expression levels of biomarkers in EMS were verified by quantitative reverse transcription polymerase chain reaction (qRT-PCR) and western blotting, then further validated in the GSE5108 dataset. CIBERSORT was used to estimate the composition pattern of immune cell components in EMS.

**Results:**

One hundred and fifty-three differential expression genes (DEGs) were identified between EMS and endometrial with 83 upregulated and 51 downregulated genes. Gene sets related to arachidonic acid metabolism, cytokine–cytokine receptor interactions, complement and coagulation cascades, chemokine signaling pathways, and systemic lupus erythematosus were differentially activated in EMS compared with endometrial samples. Aquaporin 1 (AQP1) and ZW10 binding protein (ZWINT) were identified as diagnostic markers of EMS, which were verified using qRT-PCR and western blotting and validated in the GSE5108 dataset. Immune cell infiltrate analysis showed that *AQP1* and *ZWINT* were correlated with M2 macrophages, NK cells, activated dendritic cells, T follicular helper cells, regulatory T cells, memory B cells, activated mast cells, and plasma cells.

**Conclusion:**

*AQP1* and *ZWINT* could be regarded as diagnostic markers of EMS and may provide a new direction for the study of EMS pathogenesis in the future.

## Introduction

Endometriosis (EMS) is defined as the presence of ectopic endometrial glands and stroma outside of the uterine cavity and affects 6–10% of reproductive-aged women. Women with EMS can have symptoms of dyspareunia, dysmenorrhea, irregular uterine bleeding, and chronic pelvic pain [1–3]. Although medical therapies can relieve symptoms in up to 50–80% of cases, residual symptoms are still present in at least 20% of patients [4–7]. Decreased quality of life, increased surgical intervention, and increased use of assisted reproductive technology caused by EMS result in high social costs [8]. Thus, EMS has become a critical social problem that needs to be addressed.

EMS is similar to malignancies in certain respects. Both show estrogen-dependent growth, invasive growth and progression, and recurrence, and both have a tendency to metastasize [9, 10]. EMS can be divided into four disease stages on the basis of the amount, severity, location, and depth or size of growths: minimal disease (stage I), mild disease (stage II), moderate disease (stage III), and severe disease (stage IV) [11, 12]. Furthermore, EMS infiltration of more than 5 mm under the peritoneum is defined as deep EMS (DE) [13]. However, this classification cannot be used to predict clinical outcomes, symptomatology, or pain [14]. Medical professionals dealing with EMS face many issues regarding the diagnosis, treatment, and follow-up of patients, and EMS has the highest incidence rate among benign gynecological disorders in premenopausal women [15, 16]. External endometrial lesions are common in the pelvic peritoneum and ovary, and can also be found in abdominal scars, bladder, ureter, intestines, and appendix, but are rare in the brain and eye [4, 5]. DE is a nodular form that can coexist in the pelvis [17, 18]. Therefore, it is very difficult to treat when EMS invades the surrounding organs such as the bladder or rectum. Most deep rectovaginal lesions are thought to originate from the posterior part of the cervix, followed by infiltration into the anterior wall of the rectum [19–21]. The invasion process dominated by collective cell migration is considered the most invasive form of DE [22, 23]. Adenomyosis of cervix may be the cause of DE, which has been verified from the invasion of the cervix (lesion center) to the rectum (lesion front) [24]. In fact, collective cell migration and epithelial–mesenchymal transformation may be closely related to the pathogenesis of endometriotic nodules and adenomyosis [25].

Although there are increasing numbers of studies on EMS immune regulation, its specific mechanism remains unclear. In our paper, two EMS microarray datasets were downloaded from the Gene Expression Omnibus (GEO) database and merged them into a meta-data cohort. A DEG screen was performed comparing EMS with control (normal endometrium) data. Diagnostic biomarkers of EMS were filtered and identified with machine-learning algorithms. Another cohort was used to identify and validate candidate genes that were closely related to immune cell infiltration, and then the diagnostic prediction model was constructed by a regression method. Our study is the first to use CIBERSORT to quantify the proportions of immune cells in EMS or endometrial tissues on the basis of their gene microarray data. In addition, the correlation between the infiltrating immune cells and identified biomarkers was discussed for its potential contribution to future studies.

## Materials and methods

### Microarray data

We downloaded GSE7305 (GPL570, Affymetrix Human Genome U133 Plus 2.0 Array) and GSE25628 (GPL571, Affymetrix Human Genome U133 Plus 2.0 Array) datasets from the GEO database which were provided by Hever et al. and Crispi et al. [26, 27]. The GSE7305 dataset included 10 ectopic and 10 eutopic endometria, and the GSE25628 dataset included 7 ectopic and 9 eutopic endometria. Eutopic and ectopic endometria were obtained from the same patients, and for each patient the eutopic endometrium from the uterus served as the control for their ectopic endometrial sample. According to each dataset probe annotation file, the probes were changed into gene symbols. Because one gene symbol corresponded to multiple probes, the final expression value of the gene was considered according to the average value of the probe. The batch effect was removed with the combat function of the surrogate variable analysis package of R software [28]. Furthermore, the GSE5108 dataset (Illumina HumanWG-6 v3.0 expression beadchip) contained 11 eutopic and 11 ectopic endometria and was used as the validation cohort which was provided by Eyster et al. [29].

### DEG screening and data processing

GSE7305 and GSE25628 were merged into one meta-data cohort, while batch effects were preprocessed and removed with the combat function of the surrogate variable analysis package. The background correction, endometrial uniformity, and differential expression analysis between arrays was performed with the limma package of R. *P* < 0.05 and log fold change > 2 was regarded as the critical cutoffs for DEGs.

### Functional enrichment analysis

Disease Ontology (DO), Gene Ontology (GO) and KEGG pathway enrichment analyses were executed with the clusterProfiler, org.Hs.eg.db, DOSE, and enrichplot packages in R. Significant functional terms between EMS and control samples were identified with the gene set enrichment analysis (GSEA). c2.cp. kegg. v7.4. symbols. gmt was used as the reference gene set. *P* < 0.05 and a false discovery rate < 0.025 were regarded as significantly enriched.

### Candidate diagnostic biomarker screening

We used two machine-learning algorithms to identify significant prognostic variables. Least absolute shrinkage and selection operator (LASSO) is a regression analysis algorithm that was performed by the glmnet package in R to identify genes that could significantly distinguish eutopic and ectopic endometria. Another machine-learning technique that was used in our study was the support vector machine (SVM), which was widely used for classification or regression. A recursive feature elimination (RFE) algorithm was used to select the appropriate genes to avoid overfitting [30]. Then, SVM-RFE was applied to select features that identified a set of genes with the highest discriminatory power. Candidate gene expression levels were validated in the GSE5108 dataset using the two algorithms.

### Diagnostic value of featured biomarkers

We generated a receiver operating characteristic (ROC) curve using the data from 15 ectopic and 19 eutopic endometria to test the predictive value of the identified biomarkers. The diagnostic effectiveness was determined by the area under the ROC curve (AUC) value in discriminating EMS from endometria and was further validated in the GSE5108 dataset.

### Verification of the diagnostic biomarker results

Endometriosis or matched control endometrium from the same patients were obtained from Beijing Obstetrics and Gynecology Hospital, Beijing, China. We collected 45 cases were diagnosed as ovarian endometriosis cyst with the inclusion conditions: aged between 20 and 45 years, BMI of 19–25 kg/m^2^, non vegetarian patients, no operative contraindication; The exclusion criteria were diabetes and other endocrine diseases, as well as serious gastrointestinal, cardiopulmonary and liver diseases. All donors had not taken drugs and hormones before surgery, and underwent combined uterine and abdominal surgery because of abnormal uterine bleeding at the same time. All tissue samples were taken from tissues excised during surgery after being approved and informed by patients and the ethics review committee of our hospital. Protein preparation, western blotting, RNA isolation, and qRT-PCR were performed as described previously [31]. Proteins were extracted using the M-PEK kit, following the manufacturer’s instructions. The protein concentration in the extracts was determined using the Quick Start Bradford protein assay. SDS-PAGE was conducted with protein samples of approximately 20 μg loaded onto a 7% Tris–acetate gel, run at 120 V for 2 h. To maintain the integrity of the ABCA1 protein, samples were not heated prior to electrophoresis. Proteins in the gel were transferred onto an Immobilon-NC transfer membrane at 300 mA for 90 min. The membrane was blocked in 5% nonfat milk powder in Tris-buffered saline with 0.1% Tween 20 (TBST) for 2 h, incubated overnight at 4 °C with rabbit monoclonal anti-human aquaporin 1 (*AQP1*) (ab168387; Abcam) and ZW10 binding protein (*ZWINT*) (ab252950; Abcam) antibodies (1:1000 in TBST), and washed with TBST three times for 10 min each. It was then incubated for 45 min at room temperature (RT) with a goat anti-rabbit IgG and a mouse anti-rabbit IgG-horseradish peroxidase (HRP)-conjugated secondary antibody (1:5000 in TBST). After three 10-min washes of the membrane in TBST, the signal was recorded by digital imaging using ChemiDocTM XRS + with Image Lab™ Software (BIO-RAD, Hercules, California, USA). *β*-actin served as an internal control. RNA was extracted from snap-frozen placental samples. The tissues were placed in precooled TRIzol reagent (Thermo Fisher Scientific, Waltham, MA, USA), and RNA was extracted immediately using chloroform extraction and isopropanol precipitation. The amount of extracted RNA was quantified using a spectro-photometer. Reverse transcription was performed in a gradient cycler using a kit (Thermo Fisher Scientific), following the manufacturer’s instructions. The 20 μL reaction containing 2 μg of total RNA was incubated at 25 °C for 10 min and 37 °C for 120 min, followed by 85 °C for 5 min and 4 °C for 10 min. Light Cycler DNA Master SYBR Green (Roche Diagnostics, Germany) was used to perform qRT-PCR, using the sense primer 5′-AGAGGGACCCACCTTGCTAA-3′ and anti-sense 5′-GCACAAAG CAATCACCGAGG-3′ for AQP1; the sense primer 5′-AACTCCGGGAAGCCTTTGAG-3′ and anti-sense 5′-TTCTGGACTGCTCTGCGTT T-3′ for ZWINT. The amplicon length was 201 bases, and GAPDH served as the internal control. PCR reaction mixtures contained 3 mM MgCl2, 0.4 μM forward and reverse primers, and 1 μL of LightCycler DNA Master SYBR Green I (10 × concentrate, Roche). An initial denaturation step at 95 °C for 75 s was performed to activate the FastStart DNA polymerase and to ensure complete denaturation of the cDNA before amplification. ZWINT, AQP1 and GAPDH were amplified during 40 cycles of denaturation at 95 °C for 15 s, annealing at 62 °C for 10 s, and elongation at 72 °C for 25 s. To remove nonspecific signals before SYBR Green I quantification, an elevated temperature fluorescence acquisition point, consisting of 80 °C for 3 s for ZWINT, AQP1 and 85 °C for 3 s for GAPDH, was added to each amplification cycle. After the final cycle, the amplified products were subjected to melting curve analysis to verify their integrities. The data were analyzed using the second derivate maximum method within the Light Cycler Relative Quantification Software. ZWINT, AQP1 values were expressed relative to those of GAPDH.

### Discovery of immune cell subtypes

Immune cell infiltration was calculated by CIBERSORT to quantify the relative proportion of infiltrating immune cells in EMS. The R software package corrplot was used to analyze and visualize 21 types of invasive immune cells, and the R software package vioplot was used to construct a violin diagram to visualize the differences in immune cell infiltration between the two groups.

### Correlation between identified genes and infiltrating immune cells

We used the Spearman’s rank correlation analysis in R to explore the correlations between the identified gene biomarkers and the levels of infiltrating immune cells. The chart technique with the ggplot2 package was used to visualize the identified associations.

### Statistical analysis

R 4.1.1 was used to conduct the statistical analyses. We used Student’s *t*-test or Mann–Whitney U-test to undertake group comparisons for continuous variables of endometrium-distributed variables. SVM algorithm was performed by the e1071 package in R, while the LASSO regression analysis was performed by the glmnet package. The diagnostic efficacy of the biomarkers was determined by ROC curve analysis. We used Spearman’s correlation to analyze the correlation between infiltrating immune cells and gene biomarkers. Two-tailed tests with *P* < 0.05 were regarded as statistically significant.

## Results

### Identification DEGs for EMS

**Fig. 1:**
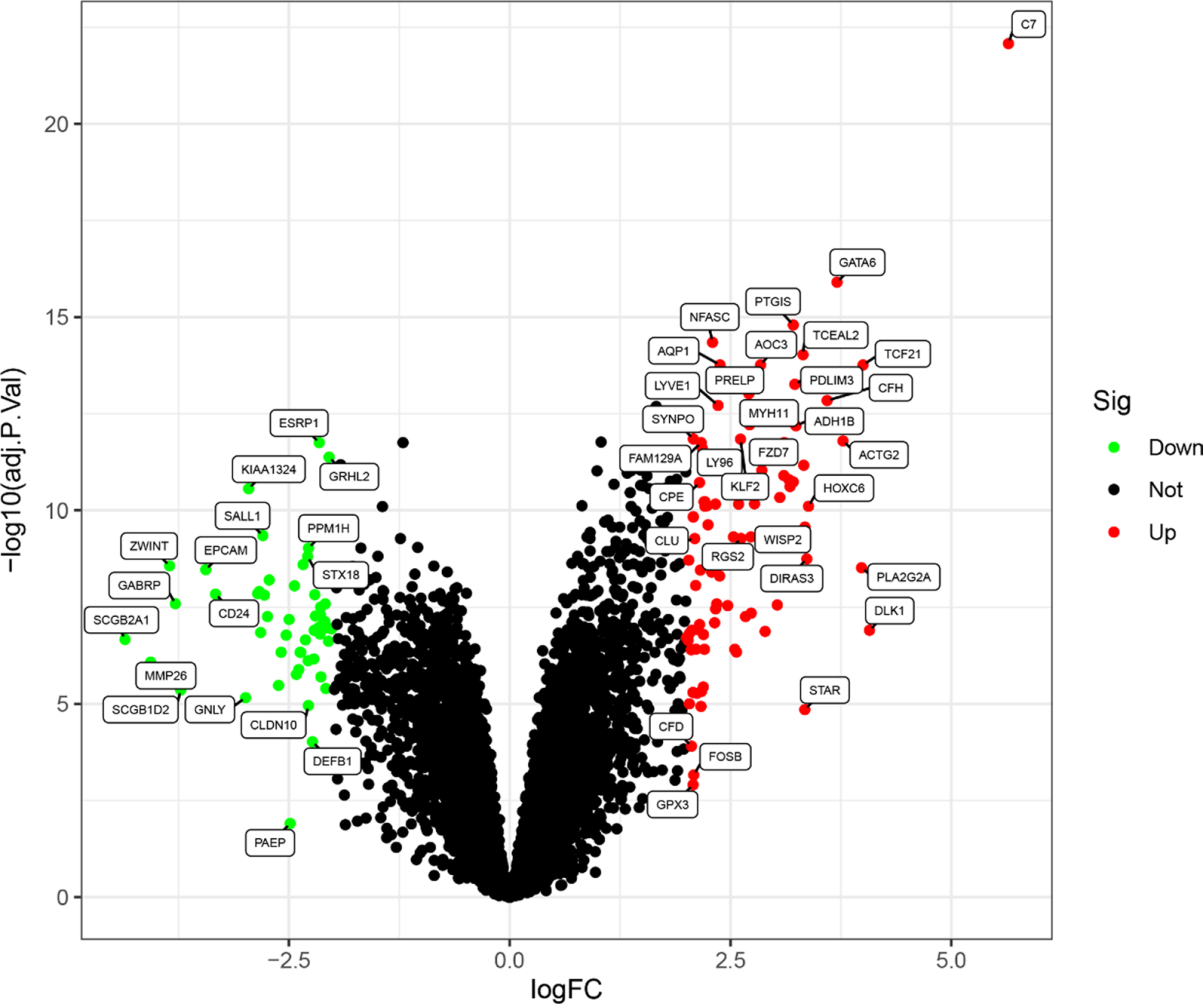
The volcano map of DEGs between EMS and endometrial samples. Each dot represents a gene, and the blue and red dots represent significantly differentially expressed genes. The red dot indicates that the gene expression is up-regulated and the blue dot indicates that the gene expression is down regulated, and the gray dot indicates that there is no significant difference between these genes in EMS

The data of 15 EMS and 19 endometria from GSE7305 and GSE25628 were retrospectively analyzed in our study. Limma package was used to analyze the differences between the EMS and endometria and the DEGs from the meta-data after the batch effects had been removed. Finally, 134 DEGs were obtained: 83 significantly upregulated genes and 51 significantly downregulated genes (Fig. 1).

### Analysis of functional correlations

**Fig. 2:**
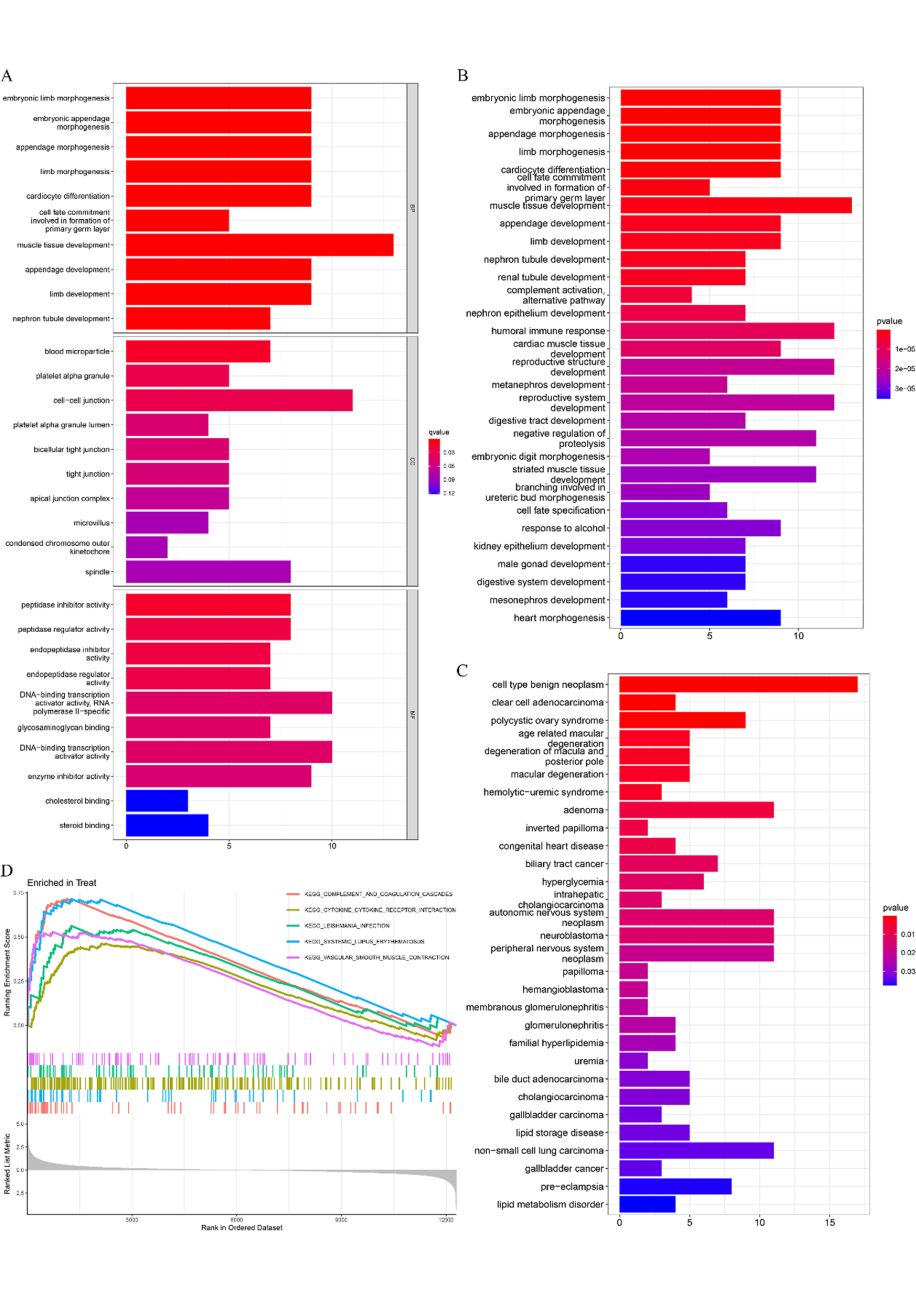
GO, KEGG, DO, and GSEA pathway enrichment. **A** GO enrichment analysis. **B** KEGG enrichment analysis. **C** DO enrichment analysis. **D** GSEA enrichment analysis

The function of the DEGs was investigated by GO, KEGG, and DO pathway enrichment analyses. We found that GO enriched by DEGs was mainly associated with muscle tissue development, embryonic limb morphogenesis, embryonic appendage morphogenesis, appendage morphogenesis, limb morphogenesis and so on (BP), cell–cell junction, blood micropaticle, platelet alpha granule and so on (CC), peptidase inhibitor activity, peptidase regulator activity, endopeptidase inhibitor activity and so on (MF), and all the correlations are statistically significant (Fig. 2A). Enriched KEGG pathways were mainly associated with primary germ layer muscle tissue development, embryonic limb morphogenesis, embryonic appendage morphogenesis, appendage morphogenesis, and so on (Fig. 2B). Moreover, the enriched diseases were mainly associated with cell type benign neoplasm, polycystic ovary syndrome, hemolytic-uremic syndrome, clear cell adenocarcinoma, and so on (Fig. 2C). The GSEA-enriched pathways were mainly associated with complement and coagulation cascades, cytokine–cytokine receptor interactions, leishmania infection, systemic lupus erythematosus, and vascular smooth muscle contraction (Fig. 2D). The above results show that the immune response is an essential part of the pathogenesis of EMS.

### Identification and validation of diagnostic biomarkers

**Fig. 3:**
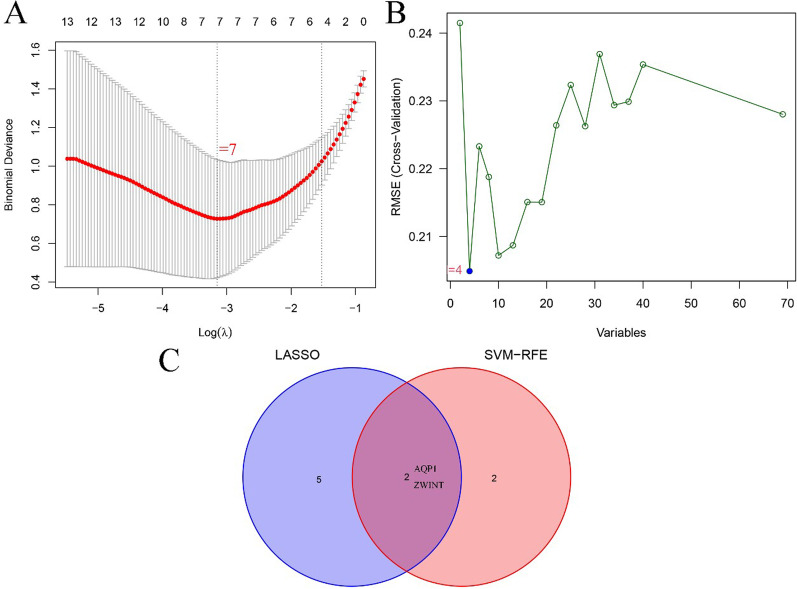
The process of screening diagnostic biomarker candidates for endometriosis. **A** Results of the LASSO regression algorithm of the DEGs for EMS. **B** Results of the SVM-RFE algorithm among the DEGs. **C** Venn diagram of the two different algorithms

**Fig. 4:**
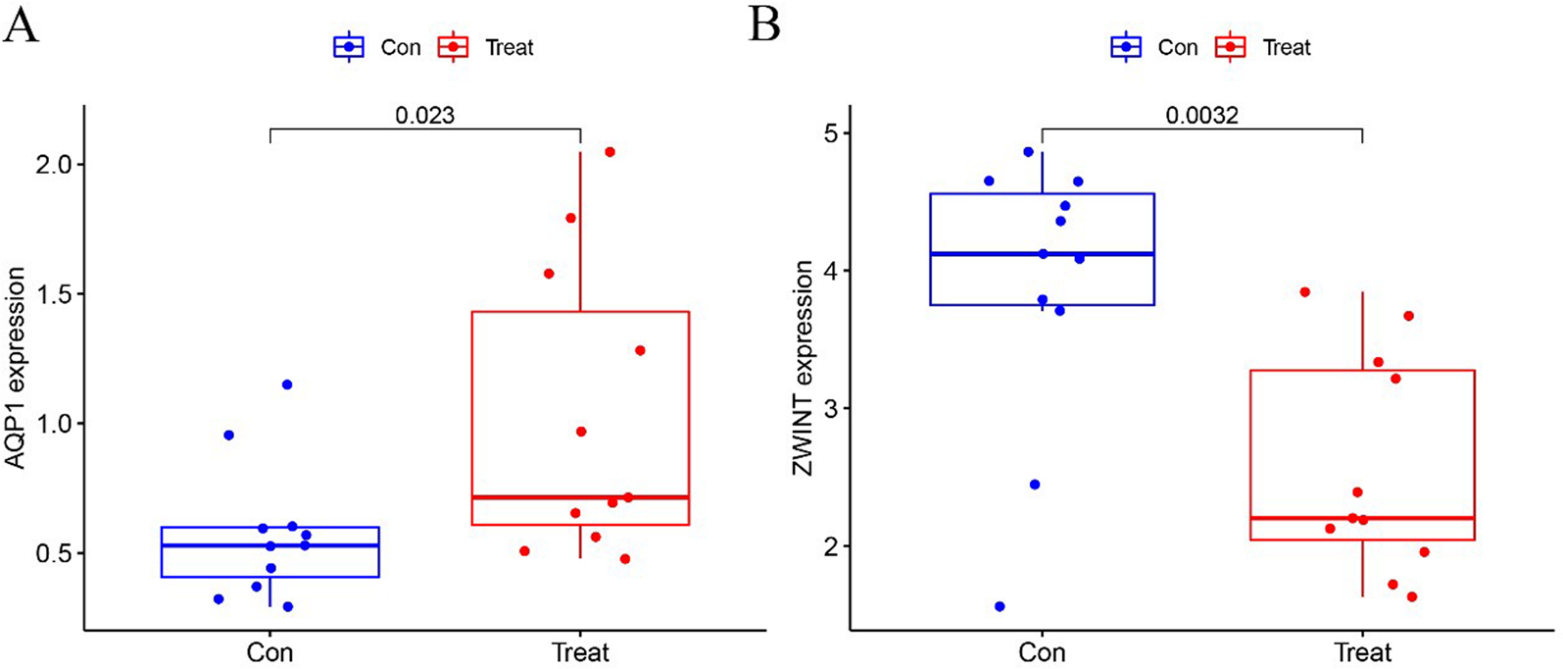
Expression of the two biomarkers in the GSE5108 dataset. **A** *AQP1*. **B** *ZWINT*

Potential biomarkers of EMS were screened by two different algorithms. Seven diagnostic biomarkers were identified by the LASSO regression algorithm of the DEGs for EMS (Fig. 3A). We determined four features among the DEGs using the SVM-RFE algorithm (Fig. 3B). The overlapping region obtained by the two calculation methods included the two screened genes (*AQP1* and *ZWINT*) (Fig. 3C). Then, the levels of the two features were verified in the GSE5108 dataset. The level of *AQP1* in EMS tissues was notably higher than that in the control group, while the level of *ZWINT* was the opposite (all *P* < 0.05; Fig. 4A, B). Finally, a logistic regression algorithm was used to establish a diagnostic model with the two identified genes.

### Verification of differential gene expression results

**Fig. 5:**
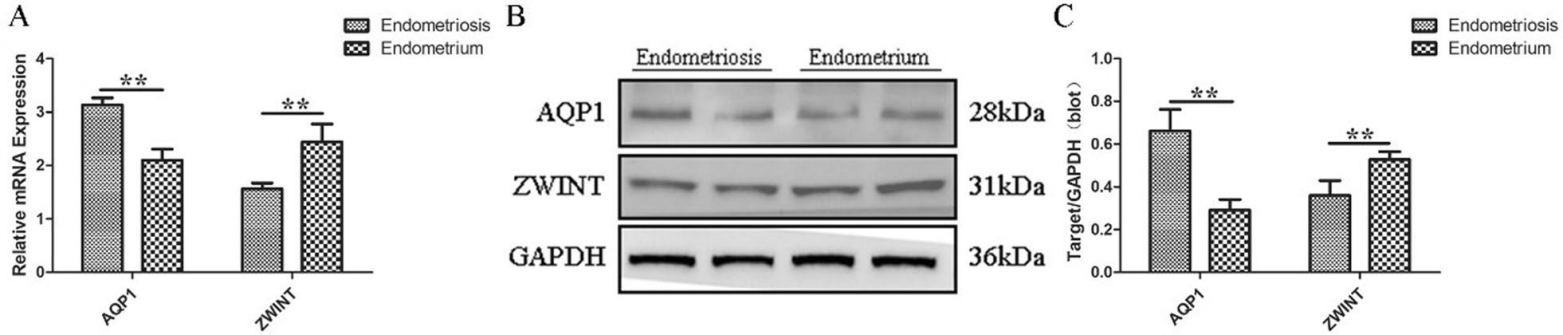
Results of differential gene expression of AQP1 and ZWINT at the mRNA and protein level between EMS and control samples. **A**. RT-PCR results; **B**. Western blotting results; **C**. Densitometry of the western blot; **P < 0.01

We verified the results of the screened differential genes at the mRNA and protein level. The verification results revealed that the mRNA (Fig. 5A) and protein (Fig. 5B, C) levels of AQP1 in EMS were higher than those in the control group, while the results of ZWINT were the opposite, consistent with the Gene Chip data with multiple exposures. The samples derive from the same experiment and those gels/blots were processed in parallel.

### Diagnostic effect of characteristic EMS biomarkers

**Fig. 6:**
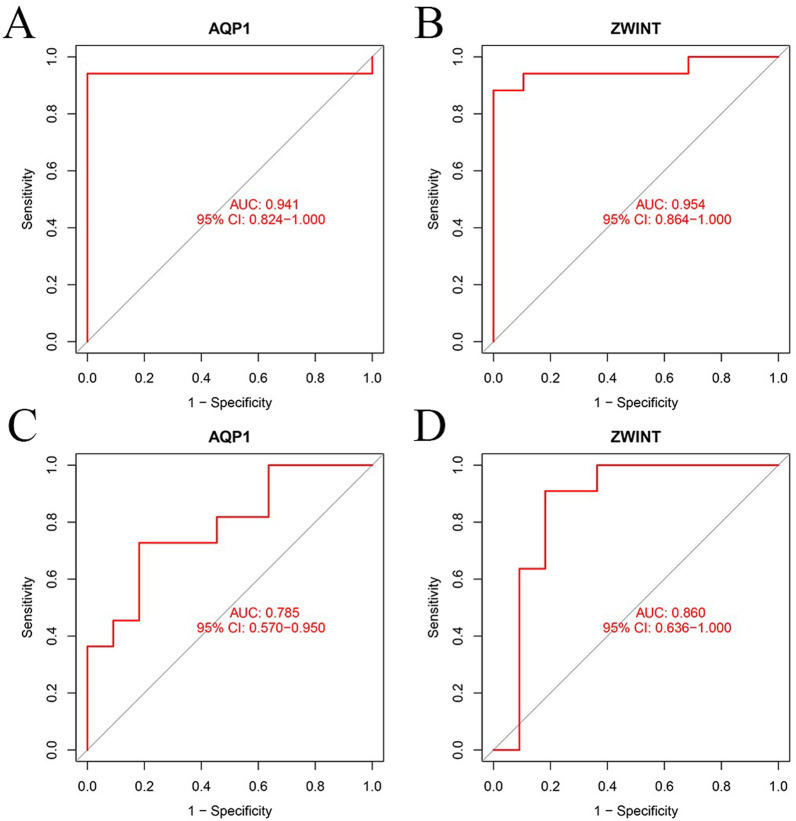
The diagnostic effectiveness of the two markers was represented by the receiver operating characteristic (ROC) curve. **A** *AQP1*. **B** *ZWINT*. **C** *AQP1* in the GSE5108 dataset. **D** *ZWINT* in the GSE5108 dataset

The diagnostic ability of the two biomarkers in discriminating EMS demonstrated a favorable diagnostic efficiency, with an AUC of 0.941 (95% confidence interval (CI) 0.824–1.000) for *AQP1* and an AUC of 0.954 (95% CI 0.864–1.000) for *ZWINT* (Fig. 6A, B). In addition, a powerful discriminatory ability was demonstrated in the GSE5108 dataset with an AUC of 0.785 (95% CI 0.570–0.950) for *AQP1* and an AUC of 0.860 (95% CI 0.636–1.000) for *ZWINT* (Fig. 6C, D). The above results indicated that the featured biomarkers had a high diagnostic ability in EMS.

### Immune cell infiltration

**Fig. 7:**
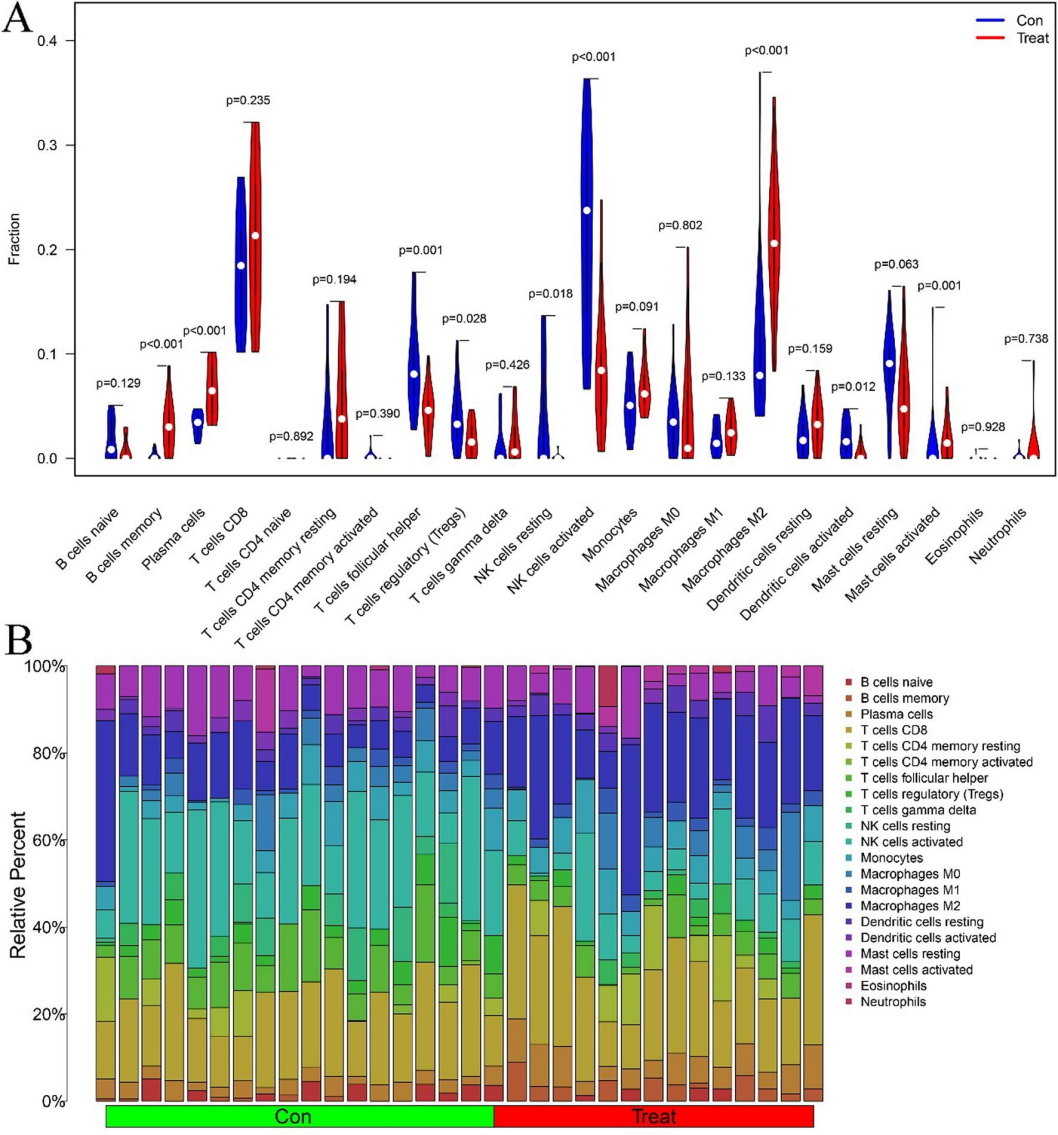
Visualization and distribution of immune cell infiltrates. **A** Composition of immune cells in the EMS and control groups. Blue and red colors represent control and EMS samples. **B** Correlation matrix results of the immune cells

First, we studied the composition of immune cells in the EMS and control groups. The proportions of T follicular helper cells (*P* = 0.001), regulatory T cells (Tregs) (*P* = 0.028), activated NK cells (*P* < 0.001), resting natural killer (NK) cells (*P* = 0.018), and activated dendritic cells (*P* = 0.012) were significantly lower in EMS tissues than in endometrial tissues. Furthermore, the proportion of memory B cells (*P* < 0.001), activated mast cells (*P* = 0.001), M2 macrophages (*P* < 0.001) and plasma cells (*P* < 0.001) in EMS was evidently higher than that in endometrial tissues (Fig. 7A).

The correlations of 21 types of immune cells were analyzed (Fig. 7B). Eosinophils were positively correlated with activated NK cells, T follicular helper cells, and M1 macrophages, but negatively correlated with M2 macrophages, activated dendritic cells, and memory B cells. T follicular helper cells were positively correlated with Tregs and activated NK cells but negatively correlated with M2 macrophages. Activated NK cells were positively correlated with T follicular helper cells but negatively correlated with plasma cells, M2 macrophages, and memory B cells. Activated memory CD4 T cells were positively correlated with resting activated NK cells but negatively correlated with M2 macrophages and monocytes. Resting mast cells were positively correlated with activated NK cells but negatively correlated with activated mast cells and M0 macrophages. Resting NK cells were positively correlated with activated dendritic cells, Tregs, and activated memory CD4 T cells but negatively correlated with M2 macrophages. Activated dendritic cells were significantly positively correlated with activated memory CD4 T cells and resting NK cells but significantly negatively correlated with plasma cells, resting dendritic cells, monocytes, and M1 macrophages. Naive B cells were positively correlated with Tregs, activated NK cells, and T follicular helper cells but negatively correlated with memory B cells and plasma cells. Neutrophils were significantly positively correlated with activated M1 macrophages, M0 macrophages, and gamma-delta T cells but negatively correlated with resting T follicular helper cells and mast cells.

### Correlations between the biomarkers and infiltrating immune cells

**Fig. 8:**
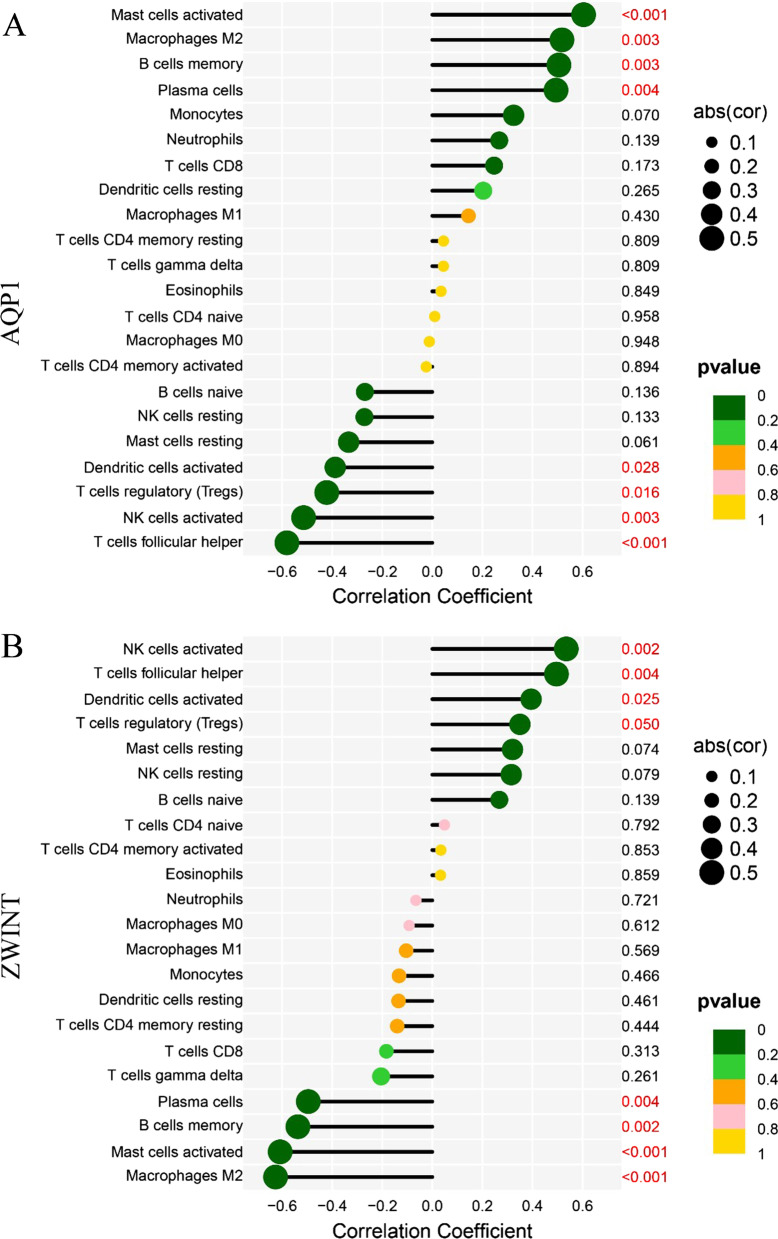
Correlation between the two biomarkers and infiltrating immune cells in endometriosis. **A** *AQP1*. **B** *ZWINT*

*AQP1* was positively correlated with activated mast cells (r = 0.6, *P* = 0.00025), M2 macrophages (r = 0.52, *P* = 0.0027), memory B cells (r = 0.51, *P* = 0.0031), and plasma cells (r = 0.49, *P* = 0.0045), and negatively correlated with follicular helper T cells (r =  − 0.58, *P* = 0.00061), activated NK cells (r =  − 0.51, *P* = 0.003), Tregs (r =  − 0.42, *P* = 0.016), and activated dendritic cells (r =  − 0.39, *P* = 0.028) (Fig. 8A). *ZWINT* was positively correlated with activated dendritic cells (r = 0.39, *P* = 0.025), activated NK cells (r = 0.54, *P* = 0.0019), T follicular helper cells (r = 0.5, *P* = 0.0043), and Tregs (r = 0.35, *P* = 0.05), and negatively correlated with M2 macrophages (r =  − 0.63, *P* = 0.00015), activated mast cells (r =  − 0.61, *P* = 0.00022), memory B cells (r =  − 0.54, *P* = 0.0015), and plasma cells (r =  − 0.5, *P* = 0.0044; Fig. 8B).

## Discussion

EMS is a progressive disease that is mainly manifested over three aspects: the gradual aggravation of dysmenorrhea, the gradual increase in EMS cysts, and the gradual increase in EMS stage. It is very important to intervene and delay its progress, but diagnosis is often delayed. The internationally agreed definition of delayed diagnosis of EMS is the interval from the onset of pain symptoms to the surgical diagnosis of EMS, and the delay time of EMS diagnosis ranges 4–10 years [32–34]. At present, the diagnostic process in EMS is generally to visit a doctor after dysmenorrhea symptoms, undergo gynecological and auxiliary examinations, and receive a diagnosis after laparoscopic surgery and postoperative pathological examination. The clinical diagnosis of EMS (non-surgical diagnosis) has a certain value, but the gold standard for EMS diagnosis is surgery and postoperative pathological examination. The reasons for the delay in the diagnosis of EMS are controversial. Studies found that the main reasons for the delay in the diagnosis of EMS are the patients' insufficient attention to dysmenorrhea, the doctors' insufficient understanding of EMS, the lack of non-invasive diagnostic methods, and the limitations of surgical diagnosis [33, 35, 36]. Although a well-trained ultrasound doctor or radiologist with expertise in the MRI manifestations of endometriosis may contribute to the diagnosis of endometriosis, the preoperative evaluation of endometriosis with pouch of Douglas obliteration using a noninvasive technique in stage III–IV endometriosis is otherwise still a major challenge [37]. Diagnosis delay is an important clinical problem affecting the diagnosis and treatment of patients with EMS. It will not only delay treatment and miss the best treatment opportunity, but also lead to the progression of EMS to a certain extent, increase the degree of pain and the probability of infertility, and also increase the difficulty and trauma of surgery. Thus, it is very important to identify early diagnostic markers that could play an important role in the diagnosis and treatment of EMS. In recent years, an increasing number of researchers have searched for new diagnostic biomarkers of EMS and explored the components of immune cell infiltrates in EMS, which may have a beneficial impact on the clinical results of EMS patients. Moreover, mRNA and microRNA have become promising biomarkers in EMS. Further, only a few studies have investigated the abnormal expression of gene biomarkers and endometrial immune infiltration in EMS. Therefore, the purpose of this study was to identify candidate diagnostic biomarkers for EMS and to study the effect of immune cell infiltration in EMS.

With the rapid development of high-throughput sequencing technology and high-resolution instruments, a large number of omics data, such as genome Research on transcriptome, metabolome and proteome began to emerge. When dealing with these high-throughput and high latitude big data, how to quickly screen the data beneficial to our research object from the massive information, overcome the problem of multicollinearity, find treatment targets and realize precision medicine has become a research hotspot today. Machine learning uses cutting-edge theories such as statistical analysis and algorithm complexity to simulate human learning methods, so as to train experience and methods from real data, continuously improve and iterate to the best state, and generate the best model. Because machine learning has excellent ability to deal with complex data, it has been widely used in dealing with biological data, showing a good prospect [38]. As far as we know, our study is the first to identify diagnostic biomarkers associated with immune cell infiltrates in EMS by mining multiple GEO datasets. An integrated analysis of GSE7305 and GSE25628 datasets from GEO was conducted. Fifty-three DEGs were identified, comprising 28 upregulated and 25 downregulated genes. We found that GO enrichment was mainly associated with lung development, respiratory tube development, complement activation, alternative pathway, regulation of humoral immune response of the BP, blood microparticles, myosin filament of the CC, tubulin binding, and peptidase regulator activity of the molecular function. KEGG pathway enrichment was mainly associated with drug metabolism-cytochrome P450, tyrosine metabolism, and complement and coagulation cascades. The diseases enriched were mainly associated with benign neoplasm, polycystic ovary syndrome, gallbladder carcinoma, and adenoma cell types. The GSEA-enriched pathways were mainly associated with cytokine–cytokine receptor interactions, arachidonic acid metabolism, chemokine signaling pathways, systemic lupus erythematosus, and complement and coagulation cascades. All of the results indicated that the immune response plays a crucial role in EMS.

We identified two diagnostic markers using two machine-learning algorithms. AQPs are a group of glycoproteins that selectively transport transmembrane molecules. They are found in the uterus, ovary, fallopian tube, and other parts of the female reproductive organs, and are involved in the ovulation of follicles, menstruation, and the occurrence and development of malignant tumors or benign gynecological diseases with malignant behavior. When the endometrium changes, the expression of AQP is abnormal [39]. At present, 13 members of the AQP family have been identified, AQP0–12, among which AQP1 and AQP5 are mostly related to disease [40]. AQP1, the first-discovered AQP, is a transmembrane tetramer composed of four monomers with a molecular weight of approximately 112 kDa. Narváez-Moreno et al. confirmed that the expression level of AQP1 in benign lesions was higher than that in malignant lesions [41]. Colombelli et al. showed that AQP1 is mainly distributed in vascular endothelial cells and that a decrease in AQP1 would lead to a decrease in microvessel density, indicating that AQP1 can promote angiogenesis and be used as a marker of EMS invasion [42]. ZWINT is a component of a known centromeric complex that is composed of 278 amino acids. It can specifically bind to 80 amino acid residues at the N-terminal of the ZW10 protein, and it plays an important role in regulating mitosis and chromosome movement as well as regulating the cell cycle [43, 44]. Studies have shown that ZWINT is necessary for the assembly of spindle assembly checkpoint, and this checkpoint protein is a complex formed by a variety of binding proteins connecting chromosome centromeres and spindle tubulin, which plays an important role in accurate chromosome allocation in progeny cells [45, 46]. If the checkpoint protein is defective, it can lead to chromosome aneuploidy separation and even carcinogenesis [47]. EMS is a benign disease with malignant tumor characteristics, and the levels of ZWINT in EMS are lower than those in the endometrium. Thus, we consider that ZWINT may play a crucial role in the pathogenesis of EMS, but further research is needed to explore its role and potential mechanism in the pathogenesis of endometriosis.

There is a complex and interactive regulatory imbalance between pro-inflammatory cytokines/anti-inflammatory cytokines which are regulated by sex hormones, especially estrogen, local metabolism, and pro-inflammatory cytokines and anti-inflammatory cytokines implicated in endometriosis [48–50]. So, during the menstrual cycle, endometrial-like tissue can spread outside its endometrial location, which attract cytotoxic T cells, macrophages, and NK cells [51–54]. We used CIBERSORT to evaluate the type of immune cell infiltrates in EMS and endometrial samples. The results show that many immune cell subtypes are closely related to the biological process of EMS. An increased infiltration of M2 macrophages, activated mast cells, memory B cells, and T follicular helper cells, as well as a decreased infiltration of T follicular helper cells, activated dendritic cells, activated NK cells, and Tregs, were shown to be potentially associated with the pathogenesis of EMS. And, the results showed that AQP1 and ZWINT were correlated with memory B cells, activated mast cells, M2 macrophages, T follicular helper cells, activated dendritic cells, Tregs, and activated NK cells. In fact, the various immune cells in the abdominal cavity environment improve the invasive and adhesive abilities of endometrial cells, including dendritic cells, macrophages, mast cells, NK cells, and T cells, which can lead to ectopic endometrium flowing back into the pelvic and abdominal cavities with menstrual blood [55]. Meanwhile, anti-inflammatory factors such as NK cells, macrophages, cytotoxic T lymphocyte and T cells may contribute to novel adaptive growth patterns of the ectopic endometrium by creating a tolerant endometriotic environment and having an important role in immune escape by ectopic lesions [56–59]. Subsequently, the activation of the inflammatory response promotes the secretion of cytokines and chemokines in the abdominal cavity to create a microenvironment and induce the development of ectopic endometrial tissue by promoting local angiogenesis and destroying the process of endometrial apoptosis [60]. The large amount of evidence mentioned above as well as our current results show that several types of invasive immune cells have a crucial role in EMS and should be investigated further in future studies.

In conclusion, the decision to choose a wide range of surgery, such as low anterior intestinal resection, or extensive ureteral dissection or resection, may be painful for EMS patients, who may only need simple ovarian cyst resection. ZWINT and AQP1 may be reliable predictors of the severity of endometriosis, with high specificity and sensitivity. While, this research also has some limitations. First, the biomarker and immune cell profiles of the tissues were collected from two different datasets, and hence it is important to further validate their reproducibility. Second, the total cases in the GSE5108 validation cohort were small, thus contributing to less robust results. Third, since GSE25628, GSE7305 and GSE5108 have analysed different types of endometriotic lesions, the co-analysis of them may affect the results. Finally, the function of the two biomarkers and the role of immune cell infiltrates in EMS were inferred through bioinformatics analyses, and therefore a prospective study with a larger sample size should be performed to confirm our findings.

## Data Availability

Publicly available datasets were analyzed in this study. This data can be found here: All the raw data used in this study are derived from the public GEO data portal (https://www.ncbi.nlm.nih.gov/geo/; Accession numbers: GSE7305, GSE25628, and GSE5018).

## References

[1] Burney RO, Giudice LC (2012). Pathogenesis and pathophysiology of endometriosis. Fertil Steril.

[2] Johnson NP, Hummelshoj L, Adamson GD, Keckstein J, Taylor HS, Abrao MS, Bush D, Kiesel L, Tamimi R, Sharpe-Timms KL (2015). World endometriosis society consensus on the classification of endometriosis. Hum Reprod.

[3] Rogers PA, Adamson GD, Al-Jefout M, Becker CM, D’ Hooghe TM, Dunselman GA, Fazleabas A, Giudice LC, Horne AW (2015). Research priorities for endometriosis. Reprod Sci.

[4] Bonocher CM, Montenegro ML, Rosa E, Silva JC, Ferriani RA, Meola J (2014). Endometriosis and physical exercises: a systematic review. Reprod Biol Endocrinol.

[5] Dunselman GA, Vermeulen N, Becker C, Calhaz-Jorge C, D’ Hooghe T, De Bie B, Heikinheimo O, Horne AW, Kiesel L (2014). ESHRE guideline: management of women with endometriosis. Hum Reprod.

[6] Buggio L, Barbara G, Facchin F, Frattaruolo MP, Aimi G, Berlanda N (2015). Self-management and psychological-sexological interventions in patients with endometriosis: strategies, outcomes, and integration into clinical care. Int J Womens Health.

[7] Parasar P, Ozcan P, Terry KL (2015). Endometriosis: epidemiology, diagnosis and clinical management. Curr Obstet Gynecol Rep.

[8] Vitagliano A, Noventa M, Quaranta M, Gizzo S (2016). Statins as targeted "magical pills" for the conservative treatment of endometriosis: may potential adverse effects on female fertility represent the "dark side of the same coin". A systematic review of literature. Reprod Sci.

[9] Flores I, Rivera E, Ruiz LA, Santiago OI, Vernon MW, Appleyard CB (2007). Molecular profiling of experimental endometriosis identified gene expression patterns in common with human disease. Fertil Steril.

[10] Van Gorp T, Amant F, Neven P, Vergote I, Moerman P (2004). Endometriosis and the development of malignant tumours of the pelvis. A review of literature. Best Pract Res Clin Obstet Gynaecol.

[11] Giudice LC (2010). Clinical practice endometriosis. N Engl J Med.

[12] Mehedintu C, Plotogea MN, Ionescu S, Antonovici M (2014). Endometriosis still a challenge. J Med Life.

[13] Fauconnier A, Chapron C, Dubuisson JB, Vieira M, Dousset B, Bréart G (2002). Relation between pain symptoms and the anatomic location of deep infiltrating endometriosis. Fertil Steril.

[14] Ferrero S (2015). Endometriosis: modern management of an ancient disease. Eur J Obstet Gynecol Reprod Biol.

[15] Fukunaga M (2001). Uterus-like mass in the uterine cervix: superficial cervical endometriosis with florid smooth muscle metaplasia. Virchows Arch.

[16] Mounsey AL, Wilgus A, Slawson DC (2006). Diagnosis and management of endometriosis. Am Fam Physician.

[17] Nisolle M, Donnez J (1997). Peritoneal endometriosis, ovarian endometriosis, and adenomyotic nodules of the rectovaginal septum are three different entities. Fertil Steril.

[18] Cornillie FJ, Oosterlynck D, Lauweryns JM, Koninckx PR (1990). Deeply infiltrating pelvic endometriosis: histology and clinical significance. Fertil Steril.

[19] Donnez J, Squifflet J (2010). Complications, pregnancy and recurrence in a prospective series of 500 patients operated on by the shaving technique for deep rectovaginal endometriotic nodules. Hum Reprod.

[20] Donnez J, Jadoul P, Colette S, Luyckx M, Squifflet J, Donnez O (2013). Deep rectovaginal endometriotic nodules: perioperative complications from a series of 3298 patients operated on by the shaving technique. Gynecol Surg.

[21] Donnez J, Dolmans MM, Fellah L (2019). What if deep endometriotic nodules and uterine adenomyosis were actually two forms of the same disease. Fertil Steril.

[22] Donnez O, Soares M, Defrère S, Kerk OV, Colette S (2013). Nerve fibers are absent in disease-free and eutopic endometrium, but present in endometriotic (especially deep) lesions. J Endometr.

[23] García-Solares J, Dolmans MM, Squifflet JL, Donnez J, Donnez O (2018). Invasion of human deep nodular endometriotic lesions is associated with collective cell migration and nerve development. Fertil Steril.

[24] Orellana R, García-Solares J, Donnez J, van Kerk O, Dolmans MM, Donnez O (2015). Important role of collective cell migration and nerve fiber density in the development of deep nodular endometriosis. Fertil Steril.

[25] García-Solares J, Donnez J, Donnez O, Dolmans MM (2018). Pathogenesis of uterine adenomyosis: invagination or metaplasia. Fertil Steril.

[26] Hever A, Roth RB, Hevezi P, Marin ME, Acosta JA, Acosta H, Rojas J, Herrera R, Grigoriadis D, White E (2007). Human endometriosis is associated with plasma cells and overexpression of B lymphocyte stimulator. Proc Natl Acad Sci U S A.

[27] Crispi S, Piccolo MT, D’ Avino A, Donizetti A, Viceconte R, Spyrou M, Calogero RA, Baldi A, Signorile PG (2013). Transcriptional profiling of endometriosis tissues identifies genes related to organogenesis defects. J Cell Physiol.

[28] Leek JT, Johnson WE, Parker HS, Jaffe AE, Storey JD (2012). The sva package for removing batch effects and other unwanted variation in high-throughput experiments. Bioinformatics.

[29] Eyster KM, Klinkova O, Kennedy V, Hansen KA (2007). Whole genome deoxyribonucleic acid microarray analysis of gene expression in ectopic versus eutopic endometrium. Fertil Steril.

[30] Yu G, Wang LG, Han Y, He QY (2012). Clusterprofiler: an R package for comparing biological themes among gene clusters. OMICS J Integr Biol.

[31] Chengmao X, Li L, Yan L, Jie Y, Xiaoju W, Xiaohui C, Huimin G (2015). ABCA1 affects placental function via trophoblast and macrophage. Life Sci.

[32] Ballard K, Lowton K, Wright J (2006). What's the delay. A qualitative study of women's experiences of reaching a diagnosis of endometriosis. Fertil Steril.

[33] Hudelist G, Fritzer N, Thomas A, Niehues C, Oppelt P, Haas D, Tammaa A, Salzer H (2012). Diagnostic delay for endometriosis in Austria and Germany: causes and possible consequences. Hum Reprod.

[34] Nnoaham KE, Hummelshoj L, Webster P, D’ Hooghe T, de Cicco NF, de Cicco NC, Jenkinson C, Kennedy SH, Zondervan KT (2011). Impact of endometriosis on quality of life and work productivity: a multicenter study across ten countries. Fertil Steril.

[35] Husby GK, Haugen RS, Moen MH (2003). Diagnostic delay in women with pain and endometriosis. Acta Obstet Gynecol Scand.

[36] Mihalyi A, Gevaert O, Kyama CM, Simsa P, Pochet N, De Smet F, De Moor B, Meuleman C, Billen J, Blanckaert N (2010). Non-invasive diagnosis of endometriosis based on a combined analysis of six plasma biomarkers. Hum Reprod.

[37] Kaya C, Alay I, Guraslan H, Gedikbasi A, Ekin M, Ertaş Kaya S, Oral E, Yasar L (2018). The role of serum caspase 3 levels in prediction of endometriosis severity. Gynecol Obstet Invest.

[38] Zhu C, Jiang Z, Bazer FW, Johnson GA, Burghardt RC, Wu G (2015). Aquaporins in the female reproductive system of mammals. Front Biosci.

[39] Shen Q, Lin W, Luo H, Zhao C, Cheng H, Jiang W, Zhu X (2016). Differential expression of aquaporins in cervical precursor lesions and invasive cervical cancer. Reprod Sci.

[40] Narváez-Moreno B, Sendín-Martín M, Jiménez-Thomas G, Sánchez-Silva R, Suárez-Luna N, Echevarría M, Bernabeu-Wittel J (2019). Expression patterns of aquaporin 1 in vascular tumours. Eur J Dermatol.

[41] Colombelli KT, Santos S, Camargo A, Constantino FB, Barquilha CN, Rinaldi JC, Felisbino SL, Justulin LA (2015). Impairment of microvascular angiogenesis is associated with delay in prostatic development in rat offspring of maternal protein malnutrition. Gen Comp Endocrinol.

[42] Woo Seo D, Yeop You S, Chung WJ, Cho DH, Kim JS, Su OhJ (2015). Zwint-1 is required for spindle assembly checkpoint function and kinetochore-microtubule attachment during oocyte meiosis. Sci Rep.

[43] Wang H, Hu X, Ding X, Dou Z, Yang Z, Shaw AW, Teng M, Cleveland DW, Goldberg ML, Niu L (2004). Human Zwint-1 specifies localization of Zeste White 10 to kinetochores and is essential for mitotic checkpoint signaling. J Biol Chem.

[44] Famulski JK, Vos L, Sun X, Chan G (2008). Stable hZW10 kinetochore residency, mediated by hZwint-1 interaction, is essential for the mitotic checkpoint. J Cell Biol.

[45] Alfieri C, Chang L, Barford D (2018). Mechanism for remodelling of the cell cycle checkpoint protein MAD2 by the ATPase TRIP13. Nature.

[46] Yang Q, Cao W, Wang Z, Zhang B, Liu J (2018). Regulation of cancer immune escape: the roles of miRNAs in immune checkpoint proteins. Cancer Lett.

[47] Sotuneh N, Hosseini SR, Shokri-Shirvani J, Bijani A, Ghadimi R (2014). Helicobacter pylori infection and metabolic parameters: is there an association in elderly population. Int J Prev Med.

[48] Polyzos SA, Kountouras J, Mantzoros CS (2019). Helicobacter pylori infection and nonalcoholic fatty liver disease: are the four meta-analyses favoring an intriguing association pointing to the right direction. Metabolism.

[49] Alzahrani S, Nelson J, Moss SF, Paulus JK, Knowler WC, Pittas AG (2015). H. pylori seroprevalence and risk of diabetes: an ancillary case–control study nested in the diabetes prevention program. J Diabetes Complicat.

[50] Hansen KA (2010). Endometriosis. Clin Obstet Gynecol.

[51] Sampson JA (1927). Metastatic or embolic endometriosis, due to the menstrual dissemination of endometrial tissue into the venous circulation. Am J Pathol.

[52] Christodoulakos G, Augoulea A, Lambrinoudaki I, Sioulas V, Creatsas G (2007). Pathogenesis of endometriosis: the role of defective 'immunosurveillance'. Eur J Contracept Reprod Health Care.

[53] Dmowski WP, Ding J, Shen J, Rana N, Fernandez BB, Braun DP (2001). Apoptosis in endometrial glandular and stromal cells in women with and without endometriosis. Hum Reprod.

[54] Symons LK, Miller JE, Kay VR, Marks RM, Liblik K, Koti M, Tayade C (2018). The immunopathophysiology of endometriosis. Trends Mol Med.

[55] Mei J, Xie XX, Li MQ, Wei CY, Jin LP, Li DJ, Zhu XY (2014). Indoleamine 2,3-dioxygenase-1 (IDO1) in human endometrial stromal cells induces macrophage tolerance through interleukin-33 in the progression of endometriosis. Int J Clin Exp Pathol.

[56] Abomaray F, Gidlöf S, Götherström C (2015). Mesenchymal stromal cells are more immunosuppressive in vitro if they are derived from endometriotic lesions than from eutopic endometrium. Stem Cells Int.

[57] Slabe N, Meden-Vrtovec H, Verdenik I, Kosir-Pogacnik R, Ihan A (2013). Cytotoxic T-cells in peripheral blood in women with endometriosis. Geburtshilfe Frauenheilkd.

[58] Jeung IC, Chung YJ, Chae B, Kang SY, Song JY, Jo HH, Lew YO, Kim JH, Kim MR (2015). Effect of helixor A on natural killer cell activity in endometriosis. Int J Med Sci.

[59] Yang HL, Zhou WJ, Chang KK, Mei J, Huang LQ, Wang MY, Meng Y, Ha SY, Li DJ, Li MQ (2015). The crosstalk between endometrial stromal cells and macrophages impairs cytotoxicity of NK cells in endometriosis by secreting IL-10 and TGF-β. Reproduction.

[60] Mantovani A, Allavena P, Sica A, Balkwill F (2008). Cancer-related inflammation. Nature.

